# Percutaneous endoscopic interlaminar discectomy vs. percutaneous endoscopic transforaminal discectomy for L5/S1 lumbar disc herniation: a systematic review and meta-analysis of randomized controlled trials

**DOI:** 10.3389/fsurg.2026.1853050

**Published:** 2026-07-14

**Authors:** Huatao Chen, Cai Cheng

**Affiliations:** 1 Hebei Medical University, Shijiazhuang, Hebei, China; 2 Department of Spine Surgery I, Cangzhou Central Hospital Affiliated to Hebei Medical University, Cangzhou, Hebei, China

**Keywords:** endoscopic discectomy, L5/S1, lumbar disc herniation, meta-analysis, PEID, PETD, randomized controlled trial

## Abstract

**Background:**

L5/S1 lumbar disc herniation (LDH) presents distinctive anatomical constraints that may influence the choice of operative corridor during full-endoscopic surgery. This updated systematic review and meta-analysis compared the perioperative efficiency, clinical outcomes, and safety of percutaneous endoscopic interlaminar discectomy (PEID) and percutaneous endoscopic transforaminal discectomy (PETD) using randomized evidence focused on single-level L5/S1 LDH.

**Methods:**

We conducted a systematic review and meta-analysis in accordance with the PRISMA 2020 statement. PubMed, Embase, Cochrane CENTRAL, Web of Science, CNKI, and Wanfang were searched from inception to June 11, 2026. Randomized controlled trials with verifiable randomized allocation directly comparing PEID and PETD for single-level L5/S1 LDH were included. Continuous outcomes were synthesized as mean differences (MD), and dichotomous outcomes were synthesized as risk ratios (RR) or risk differences (RD), each with 95% confidence intervals (CI). ODI and VAS outcomes were analyzed according to specific follow-up time points. Fluoroscopy was analyzed separately as exposure duration in seconds or frequency counts. Risk of bias was assessed using RoB 2, and certainty of evidence was evaluated using GRADE.

**Results:**

Thirteen studies involving 1,059 patients were included. PEID was associated with shorter operative time than PETD (12 studies; MD = −17.07 min, 95% CI −26.05 to −8.09; I2 = 98.7%). Fluoroscopy frequency favored PEID (10 studies; MD = −8.94 counts, 95% CI −11.40 to −6.48; I2 = 99.6%), whereas fluoroscopy exposure duration reported in seconds did not show a statistically significant difference (2 studies; MD = −3.70 s, 95% CI −8.01–0.61; I2 = 99.1%). No robust between-group difference was observed for hospital stay (9 studies; MD = 0.38 days, 95% CI −0.36–1.12). VAS leg pain and ODI were generally comparable across follow-up time points. Modified MacNab excellent/good rates were also comparable between approaches (9 studies; RR = 1.01, 95% CI 0.98–1.04; I2 = 0%). Complications/adverse events did not differ significantly (9 studies; RD = 0.008, 95% CI −0.019–0.035; I2 = 0%).

**Conclusions:**

The updated evidence suggests that PEID may be associated with shorter operative time and reduced fluoroscopy frequency compared with PETD for L5/S1 LDH, but the magnitude of these perioperative effects remains uncertain because of very high between-study heterogeneity. However, postoperative pain relief, functional recovery, global clinical success, and safety outcomes appear broadly comparable. Given methodological limitations, geographic concentration of studies, and substantial heterogeneity in several outcomes, these findings should be interpreted cautiously, and procedure selection should remain individualized.

**Systematic Review Registration:**

https://www.crd.york.ac.uk/prospero/display_record.php?ID=CRD420251207128, identifier CRD420251207128.

## 1 Introduction

Lumbar disc herniation is a common degenerative spinal disorder and an important cause of low back pain and radiculopathy. Full-endoscopic lumbar discectomy has become an established minimally invasive technique for selected lumbar disc herniation and offers direct decompression with reduced soft-tissue disruption compared with conventional open approaches (1–3).

At the L5/S1 level, surgical approach selection is particularly influenced by local anatomy. The transforaminal corridor may be restricted by a high iliac crest, hypertrophic L5 transverse process, narrow foramen, facet obstruction, or a steep puncture trajectory, whereas the interlaminar window at L5/S1 is relatively wide and may provide a more direct route to intracanalicular disc fragments in selected patients (2, 4, 5).

Previous reviews have compared interlaminar and transforaminal endoscopic discectomy across broader lumbar populations or mixed study designs (9–11). However, the L5/S1 level has unique anatomical constraints, and mixed-level evidence may not directly answer the question of procedure selection for this specific segment.

The aim of this systematic review and meta-analysis was to compare PEID and PETD for single-level L5/S1 LDH using updated randomized evidence (12–24). We focused on perioperative efficiency, radiation exposure, patient-reported outcomes, global clinical success, and safety outcomes.

## 2 Methods

### 2.1 Study design

This systematic review and meta-analysis was conducted in accordance with the PRISMA 2020 statement and was prospectively registered in PROSPERO (CRD420251207128) (6). The review question was structured according to PICOS: population, adults with single-level L5/S1 lumbar disc herniation; intervention, PEID/interlaminar approach; comparator, PETD/transforaminal approach; outcomes, perioperative, pain, functional, global clinical success, and safety outcomes; and study design, randomized controlled trials.

### 2.2 Search strategy

In response to the reviewers’ comments, we updated and reconstructed the search strategy, screening records, and full-text assessment process. PubMed, Embase, Cochrane CENTRAL, Web of Science, CNKI, and Wanfang were searched from database inception to June 11, 2026. The search strategy combined terms related to lumbar disc herniation, L5/S1 or lumbosacral level, endoscopic discectomy, interlaminar and transforaminal approaches, and randomized trials. No language, publication year, or study-design restrictions were applied during the initial database search. The complete database-specific search strategies, including Boolean operators, database-specific controlled vocabulary where applicable, Chinese search terms, search dates, filters, and record counts, are provided in Supplementary Table S1.

Reference lists of eligible studies and relevant reviews were manually screened. All candidate studies, including those included in the submitted version, were reassessed according to the prespecified eligibility criteria. Therefore, the final set of included studies in the revised manuscript was determined based on the updated search results and full-text verification, rather than mechanically retaining the originally included studies.

### 2.3 Eligibility criteria

Studies were eligible if they met all of the following criteria: (1) adult patients with single-level L5/S1 lumbar disc herniation; (2) direct comparison between PEID/interlaminar and PETD/transforaminal endoscopic discectomy; (3) randomized controlled trial design with verifiable randomized allocation; and (4) at least one extractable perioperative, clinical, functional, global success, or safety outcome. Studies were excluded if they were retrospective comparative studies, quasi-randomized or nonrandomized studies, case series, duplicate or companion reports of the same study population, mixed-level LDH studies without separately extractable L5/S1 data, recurrent LDH studies, or reports without sufficient extractable data. Publicly available theses or dissertations were considered if they met the eligibility criteria and provided verifiable full-text data. Conference abstracts or trial registry records without extractable outcome data were not counted as independent studies.

### 2.4 Study selection and data extraction

Two reviewers independently screened titles and abstracts, followed by full-text assessment of potentially eligible reports. Disagreements were resolved through discussion and consensus, with consultation of a third reviewer when necessary. Full-text exclusion reasons are provided in Supplementary Table S2. Included-study characteristics are summarized in Supplementary Table S3.

Data extraction was performed independently by two reviewers using a standardized extraction form. Extracted items included first author, publication year, country, sample size, age, sex distribution, herniation subtype when available, anesthesia, surgical technique, follow-up duration, outcomes reported, and numerical outcome data. For continuous outcomes, sample size, mean, and standard deviation were extracted for the PEID/interlaminar and PETD/transforaminal groups. For dichotomous outcomes, event counts and total sample sizes were extracted.

PEID/interlaminar was defined as the intervention group and PETD/transforaminal as the comparator group; therefore, mean differences were calculated as PEID minus PETD. For Mo 2019, a publicly available master’s thesis report was used as the data source because it provided detailed full-text numerical data for an eligible randomized comparison; this was not unpublished raw data (19). In that study, outcome data were extracted using the as-treated sample sizes reported in the thesis because one patient crossed over intraoperatively due to intolerance. Bed rest time was standardized to hours. Fluoroscopy exposure was separated into exposure duration in seconds and frequency counts. ODI and VAS outcomes were analyzed according to specific follow-up time points, and repeated measurements from the same study were not pooled into an overall estimate.

Means and standard deviations were extracted directly when available. If data were reported only as medians, ranges, interquartile ranges, *P* values, or figures without extractable numerical values, they were not included in quantitative synthesis unless valid conversion was possible according to established methods. Standard deviations were not imputed arbitrarily.

### 2.5 Risk of bias assessment

The methodological quality of the included trials was assessed using the Cochrane Risk of Bias 2 (RoB 2) tool (7). Five domains were evaluated: bias arising from the randomization process, bias due to deviations from intended interventions, bias due to missing outcome data, bias in measurement of the outcome, and bias in selection of the reported result. Domain-level judgments and justifications are provided in Supplementary Table S4.

### 2.6 Statistical analysis

Continuous outcomes were synthesized as mean differences (MD), and dichotomous outcomes were synthesized as risk ratios (RR) or risk differences (RD), each with 95% confidence intervals (CI). Random-effects models were used because clinical and methodological heterogeneity was expected across studies. Statistical heterogeneity was assessed using the I2 statistic and Cochran’s *Q* test. For outcomes with substantial heterogeneity, results were interpreted cautiously and potential sources of heterogeneity were explored narratively. Meta-regression was not performed because the number of studies was limited for most outcomes and key covariates such as surgeon volume, learning-curve period, anesthesia type, and foraminoplasty details were inconsistently reported.

Sensitivity analyses were performed for studies with potential methodological or eligibility concerns. Because Luo 2015 could not be verified as a true randomized trial during final full-text reassessment, it was excluded from the final RCT-only synthesis; exploratory with-Luo analyses were retained only as robustness checks. Where appropriate, analyses excluding Shangguan 2022 were also performed for segment-specific uncertainty. Prediction intervals and sensitivity analyses are summarized in Supplementary Table S7. Publication bias was assessed qualitatively when relevant; formal funnel plot asymmetry tests were not performed when fewer than 10 studies contributed to an outcome because such tests are underpowered in that setting.

### 2.7 Certainty of evidence

The certainty of evidence for key outcomes was evaluated using the GRADE framework across the domains of risk of bias, inconsistency, indirectness, imprecision, and publication bias (8). Certainty was categorized as high, moderate, low, or very low. Downgrade decisions are detailed in Supplementary Table S5.

## 3 Results

### 3.1 Study selection and characteristics

**Figure 1:**
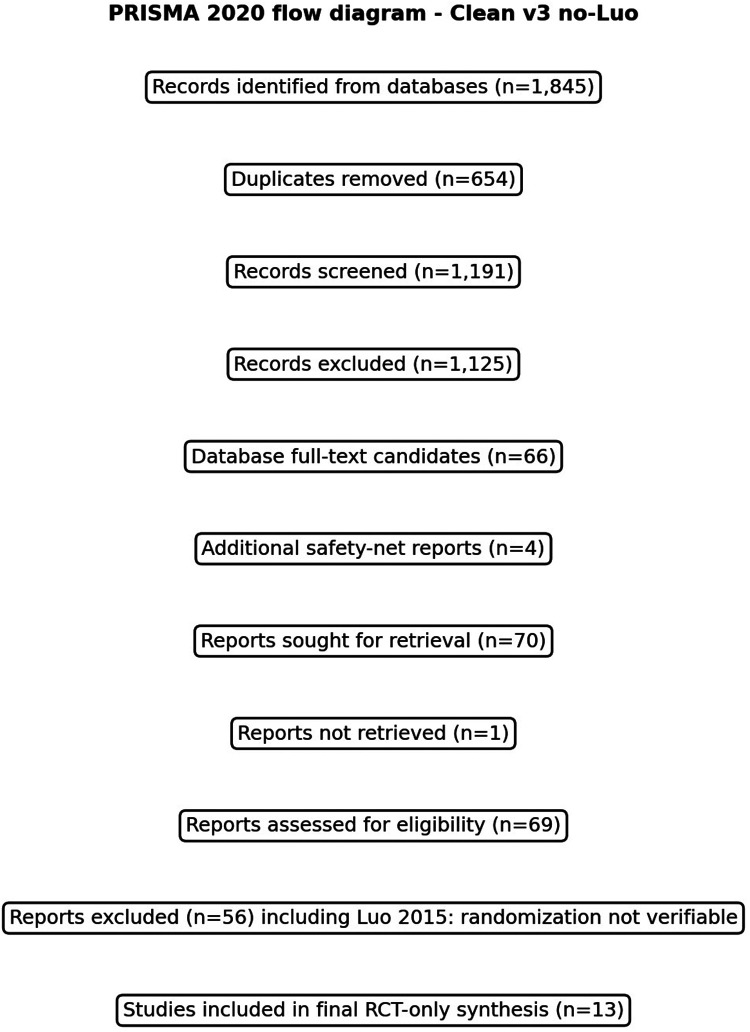
PRISMA 2020 flow diagram of study selection using the updated 66-record full-text candidate dataset.

A total of 1,845 records were identified from the updated database searches, including 342 from PubMed, 396 from Embase, 29 from Cochrane CENTRAL, 243 from Web of Science, 585 from CNKI, and 250 from Wanfang. After removing 654 duplicates, 1,191 records were screened by title and abstract. Of these, 1,125 records were excluded, leaving 66 database-screened reports for full-text assessment. In addition, four reports from the original included-study safety-net/reference-checking process were sought. Therefore, 70 reports were sought for retrieval. One report could not be retrieved despite repeated attempts, and 69 reports were assessed for eligibility. Fifty-six reports were excluded after full-text verification, and 13 studies were included in the qualitative and quantitative synthesis (Figure 1).

The 13 included studies involved 1,059 patients, including 531 in the PEID/interlaminar group and 528 in the PETD/transforaminal group (12–24). Most studies were single-center trials conducted in China. The included studies are summarized in Supplementary Table S3.

### 3.2 Risk of bias

**Figure 2:**
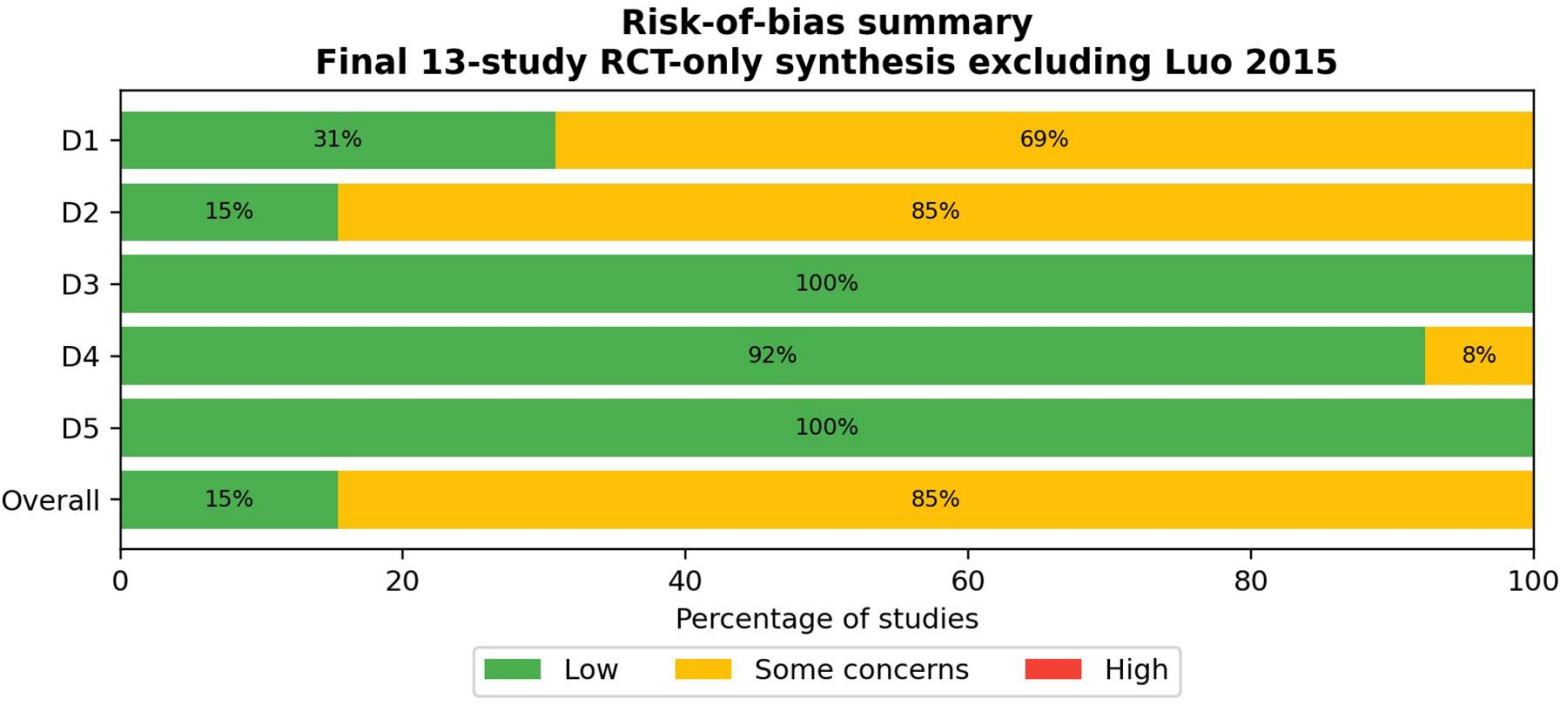
Summary of risk-of-bias judgments for the included studies based on the Cochrane RoB 2 tool.

**Figure 3:**
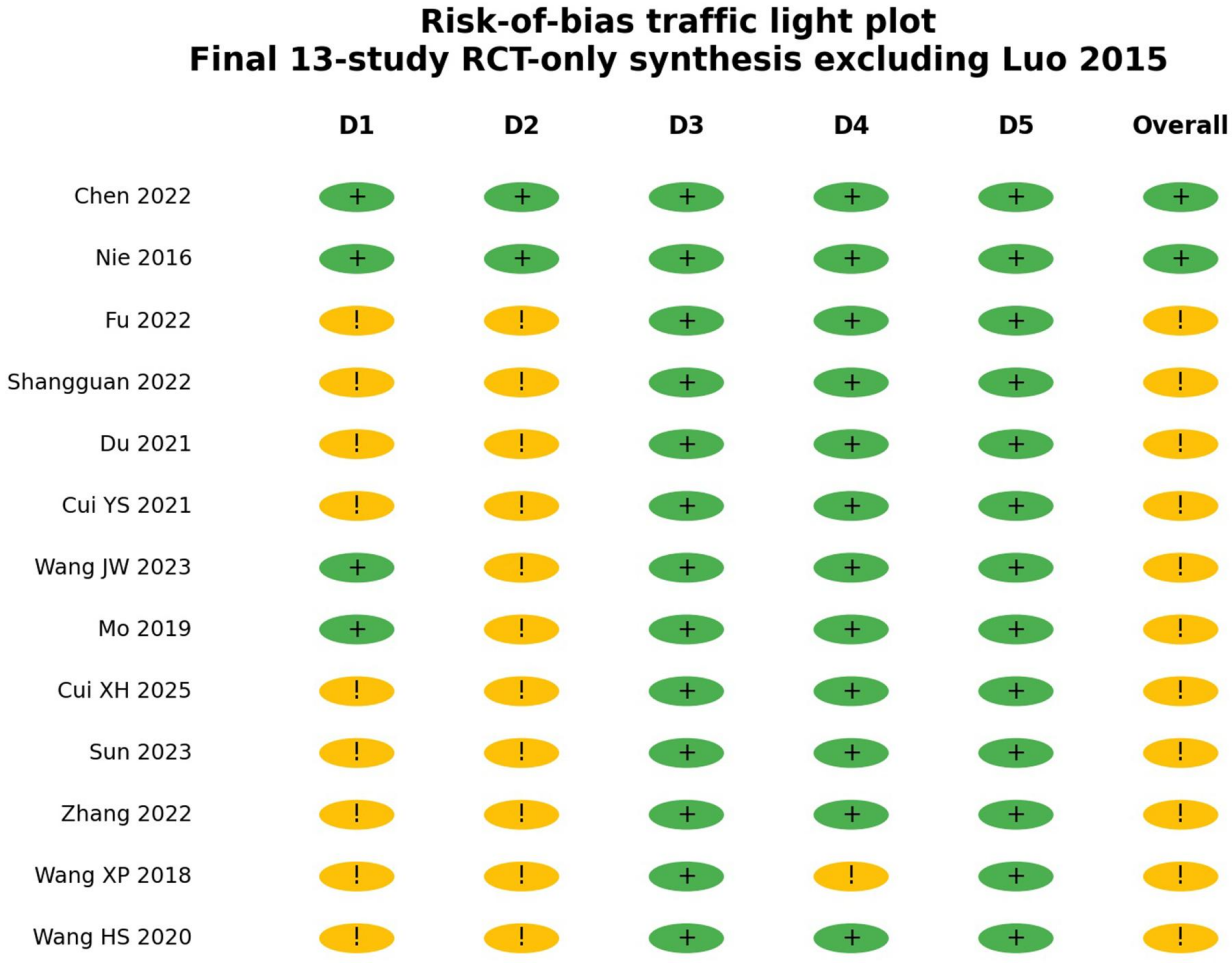
Risk-of-bias traffic light plot of the included studies assessed using the Cochrane RoB 2 tool.

Risk of bias was assessed using the RoB 2 tool (7). Domain-level judgments and justifications are provided in Supplementary Table S4 and summarized in Figures 2, 3. Several studies reported appropriate randomization methods, including computer-generated randomization, random number tables, drawing lots, or random alphabet methods. Some studies stated random allocation without describing sequence generation or allocation concealment; these trials were judged conservatively in the randomization domain. During final full-text reassessment, Luo 2015 was excluded from the RCT-only synthesis because true randomized allocation could not be verified from the full-text methods.

### 3.3 Perioperative outcomes

**Figure 4:**
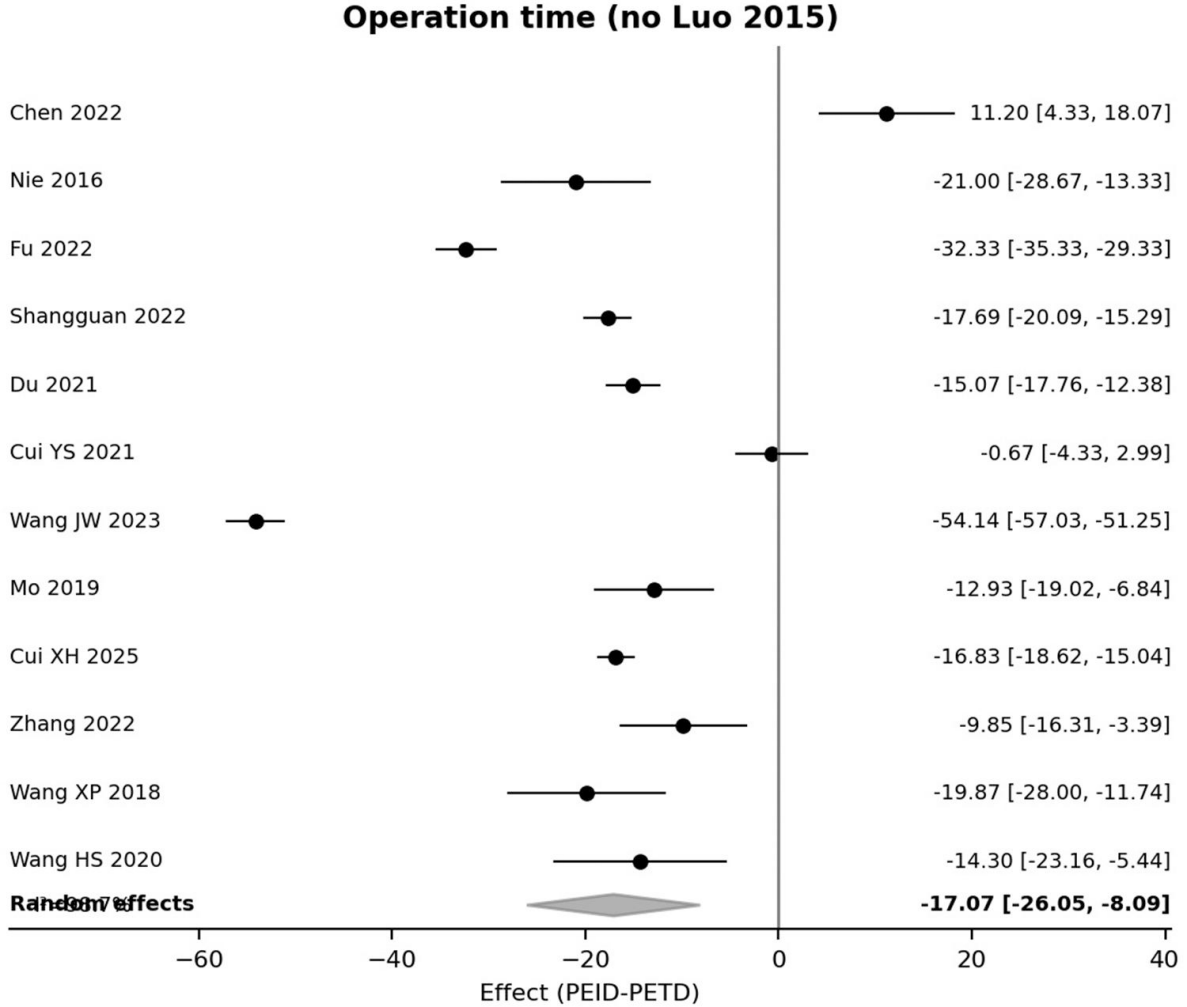
Forest plot comparing operative time between PEID and PETD for L5/S1 lumbar disc herniation.

Twelve studies reported operative time. The pooled random-effects estimate showed that PEID was associated with shorter operative time than PETD (MD = −17.07 min, 95% CI −26.05 to −8.09; I2 = 98.7%; Figure 4). Heterogeneity was substantial. Exploratory with-Luo sensitivity analysis produced a similar estimate (MD = −17.78 min, 95% CI −26.51 to −9.05; I2 = 98.6%), indicating that the overall direction of effect was not dependent on the exclusion decision.

**Figure 5:**
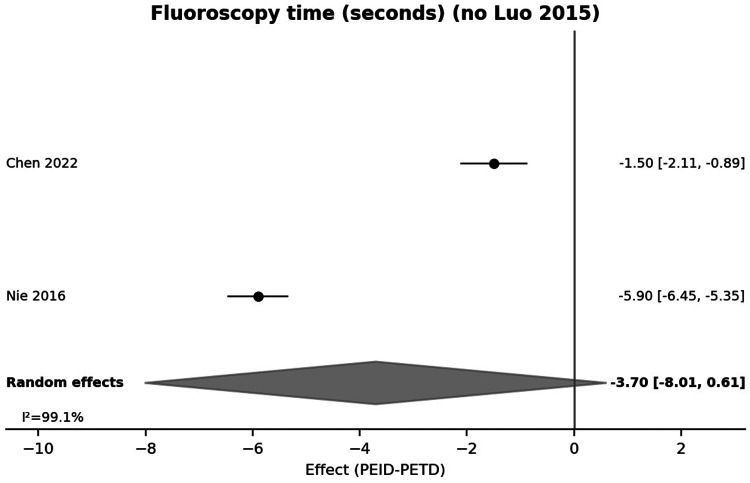
Forest plot comparing intraoperative fluoroscopy exposure duration in seconds between PEID and PETD.

**Figure 6:**
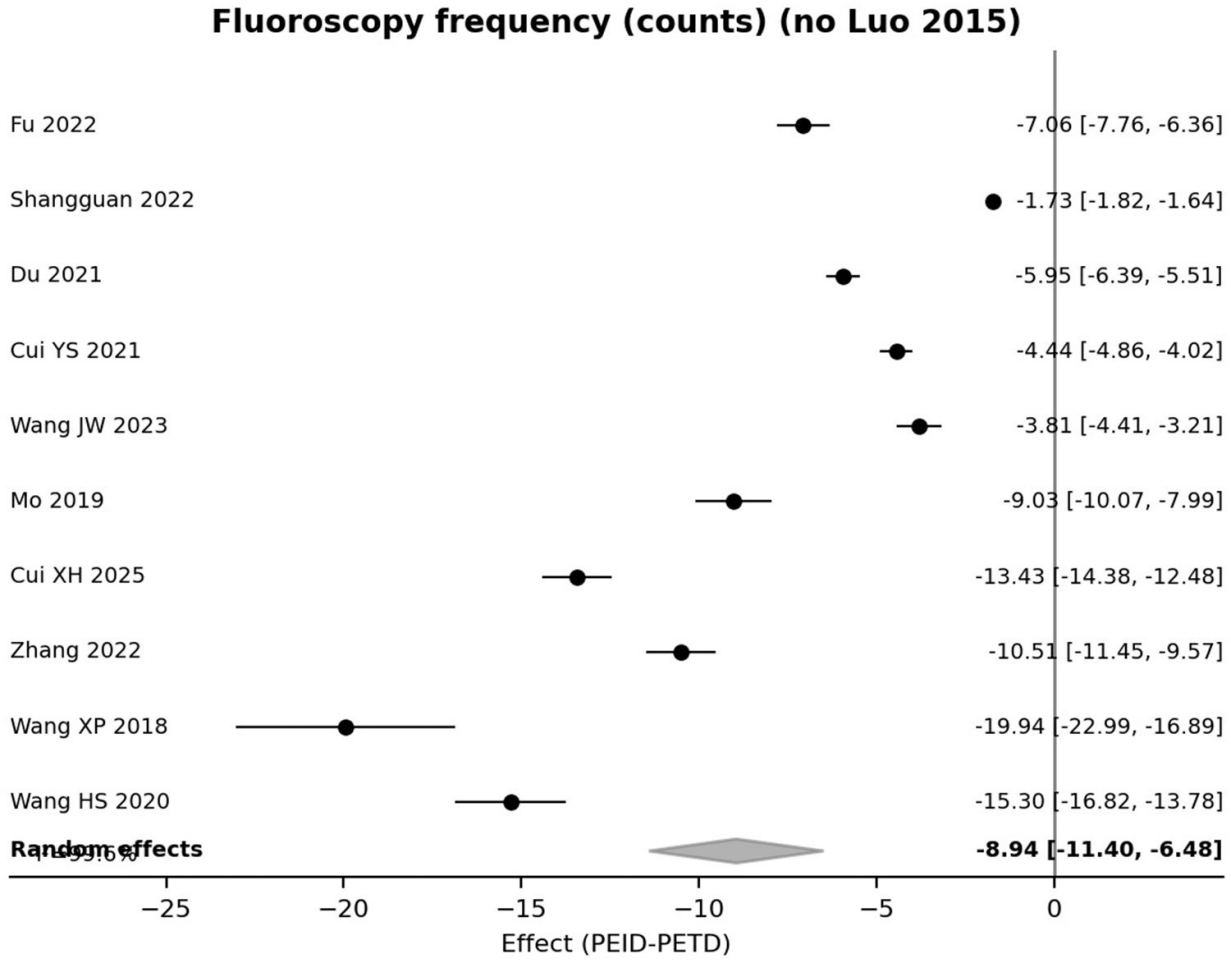
Forest plot comparing intraoperative fluoroscopy frequency in counts between PEID and PETD.

Fluoroscopy was analyzed separately according to measurement unit. Two studies reported fluoroscopy exposure duration in seconds. The pooled estimate did not reach statistical significance and showed substantial heterogeneity (MD = −3.70 s, 95% CI −8.01–0.61; I2 = 99.1%; Figure 5). Ten studies reported fluoroscopy frequency as counts. The pooled result showed fewer fluoroscopy exposures in the PEID group than in the PETD group (MD = −8.94 counts, 95% CI −11.40 to −6.48; I2 = 99.6%; Figure 6). Exploratory with-Luo sensitivity analysis did not materially change this finding (MD = −8.87 counts, 95% CI −11.23 to −6.52; I2 = 99.5%).

**Figure 7:**
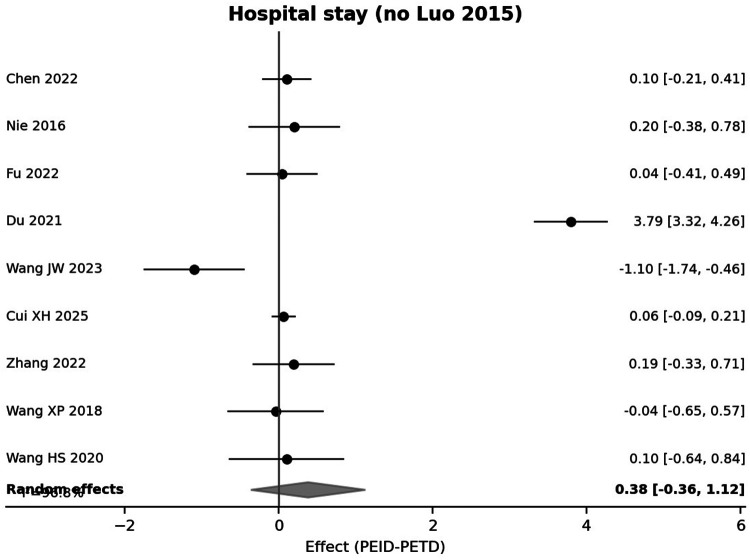
Forest plot comparing hospital stay between PEID and PETD.

**Figure 8:**
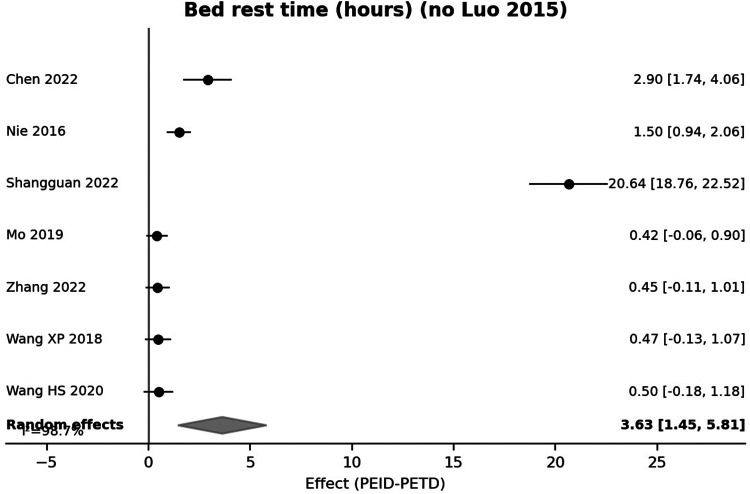
Forest plot comparing bed rest time after standardization to hours between PEID and PETD.

Nine studies reported hospital stay. No statistically significant difference was observed between PEID and PETD (MD = 0.38 days, 95% CI −0.36–1.12; I2 = 96.8%; Figure 7). Seven studies reported postoperative bed rest time after unit standardization to hours. The pooled estimate showed a longer bed rest time in the PEID group (MD = 3.63 h, 95% CI 1.45–5.81; I2 = 98.7%; Figure 8). This finding should be interpreted cautiously because postoperative mobilization protocols and discharge practices differed across studies.

### 3.4 Pain and functional outcomes

**Figure 9:**
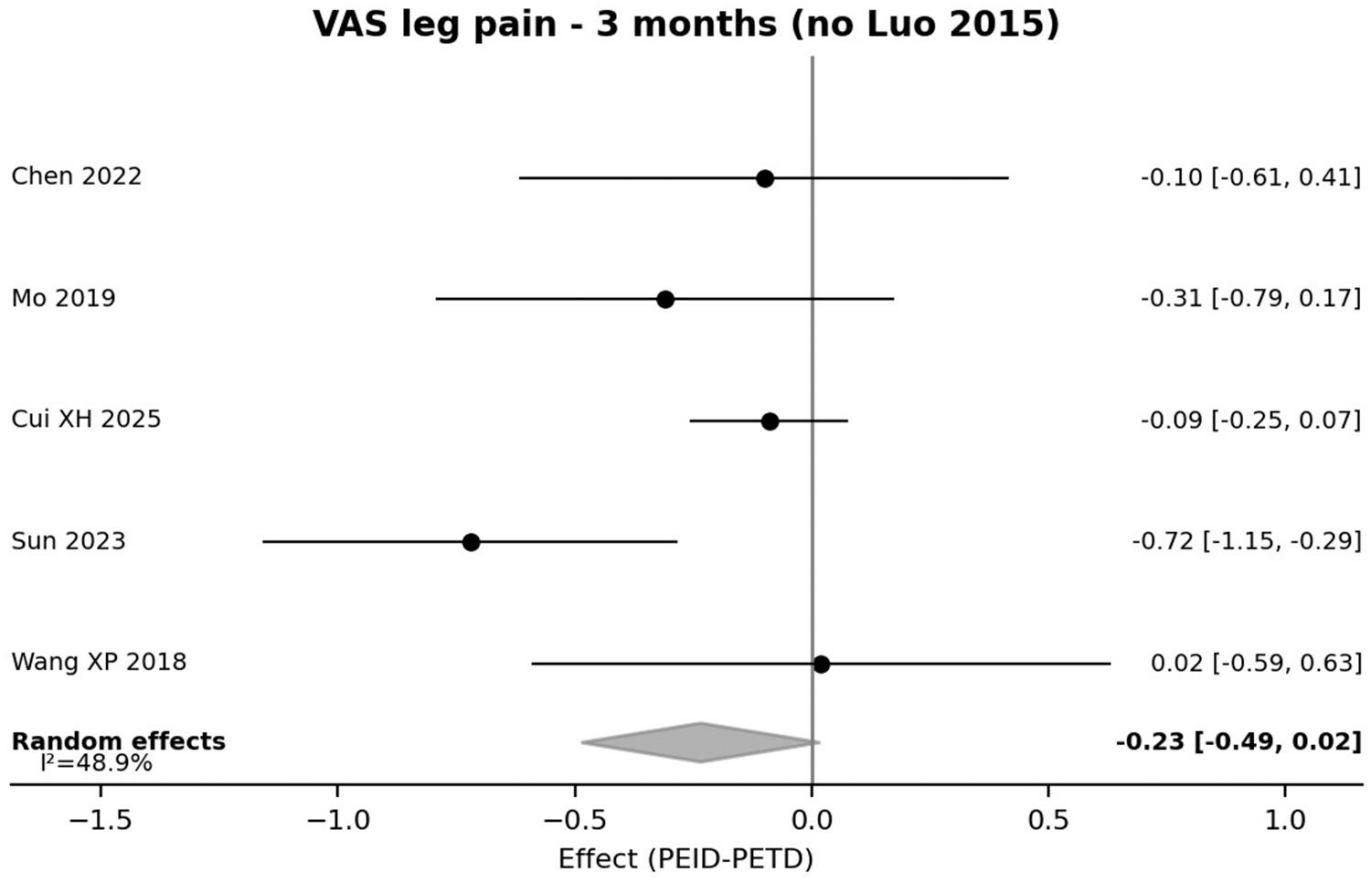
Forest plot comparing VAS leg pain scores at 3 months between PEID and PETD.

**Figure 10:**
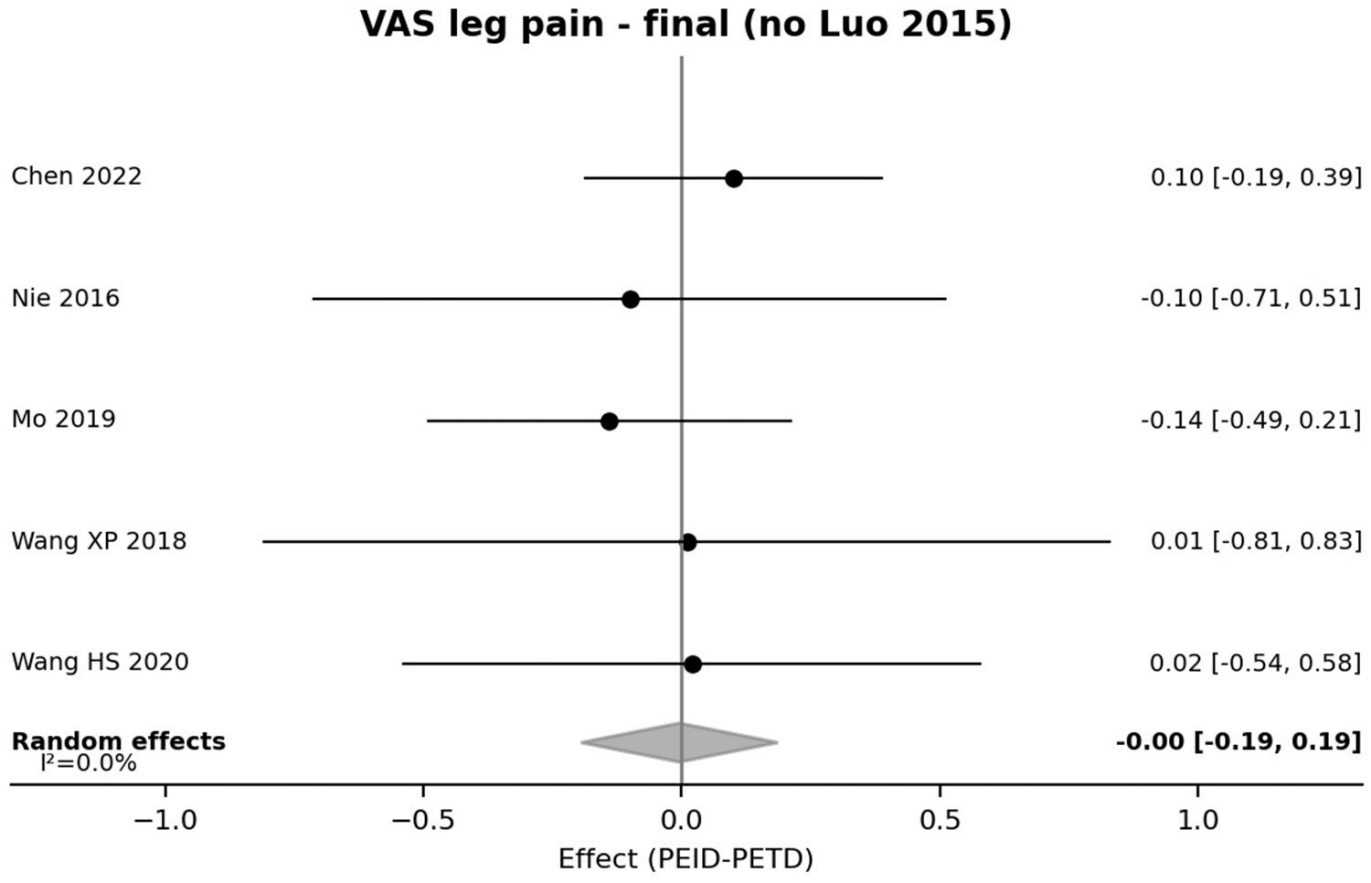
Forest plot comparing VAS leg pain scores at final follow-up between PEID and PETD.

VAS leg pain and ODI were analyzed according to follow-up time point to avoid pooling repeated measurements from the same patients. For VAS leg pain, no statistically significant difference was observed between PEID and PETD at 1 month (MD = −0.49, 95% CI −1.11–0.12; I2 = 79.2%), 3 months (MD = −0.23, 95% CI −0.49–0.02; I2 = 48.9%; Figure 9), 6 months (MD = −0.11, 95% CI −0.28–0.06; I2 = 0%), or final follow-up (MD = −0.01, 95% CI −0.20–0.18; I2 = 0%; Figure 10).

**Figure 11:**
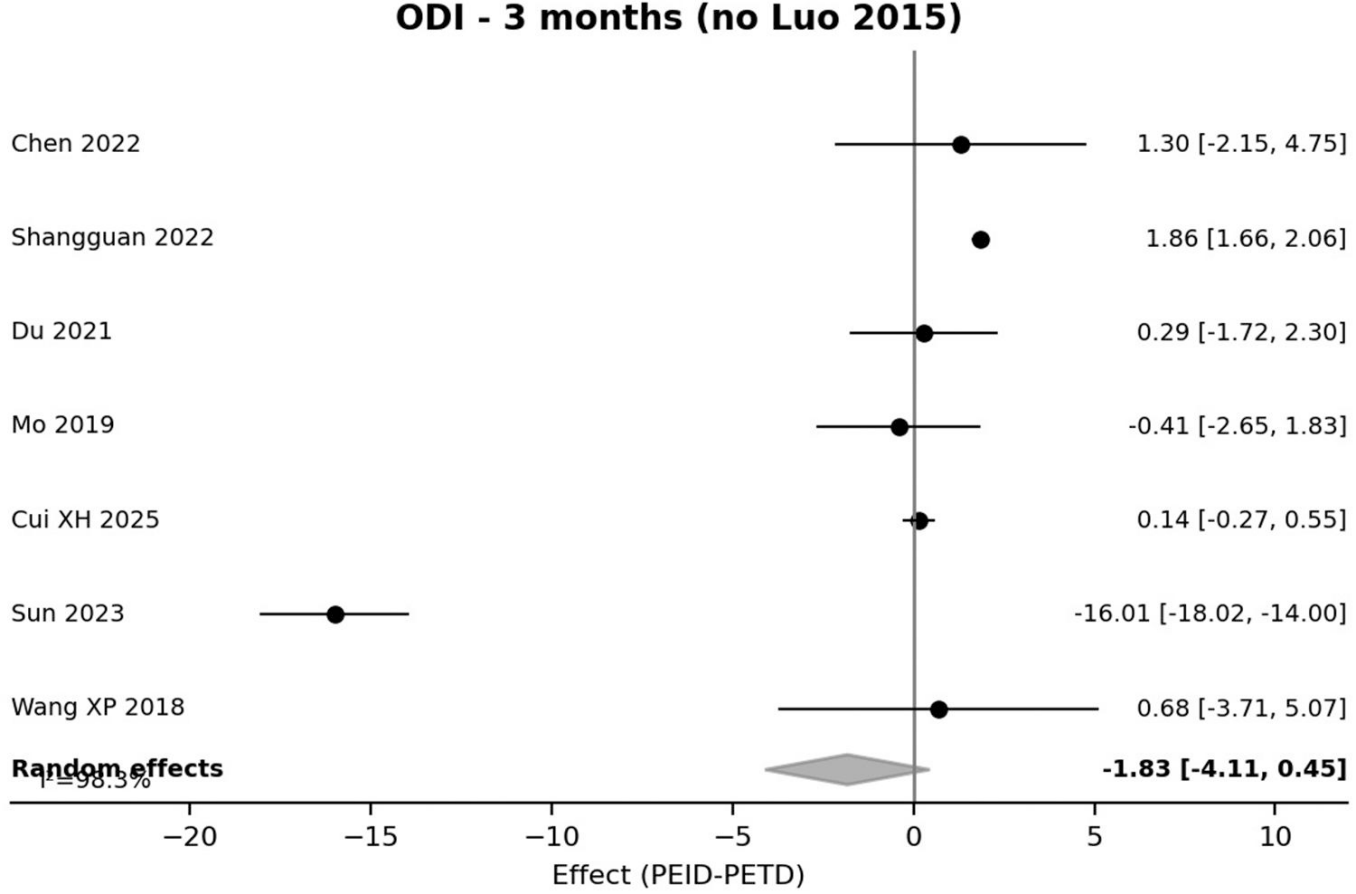
Forest plot comparing ODI scores at 3 months between PEID and PETD.

**Figure 12:**
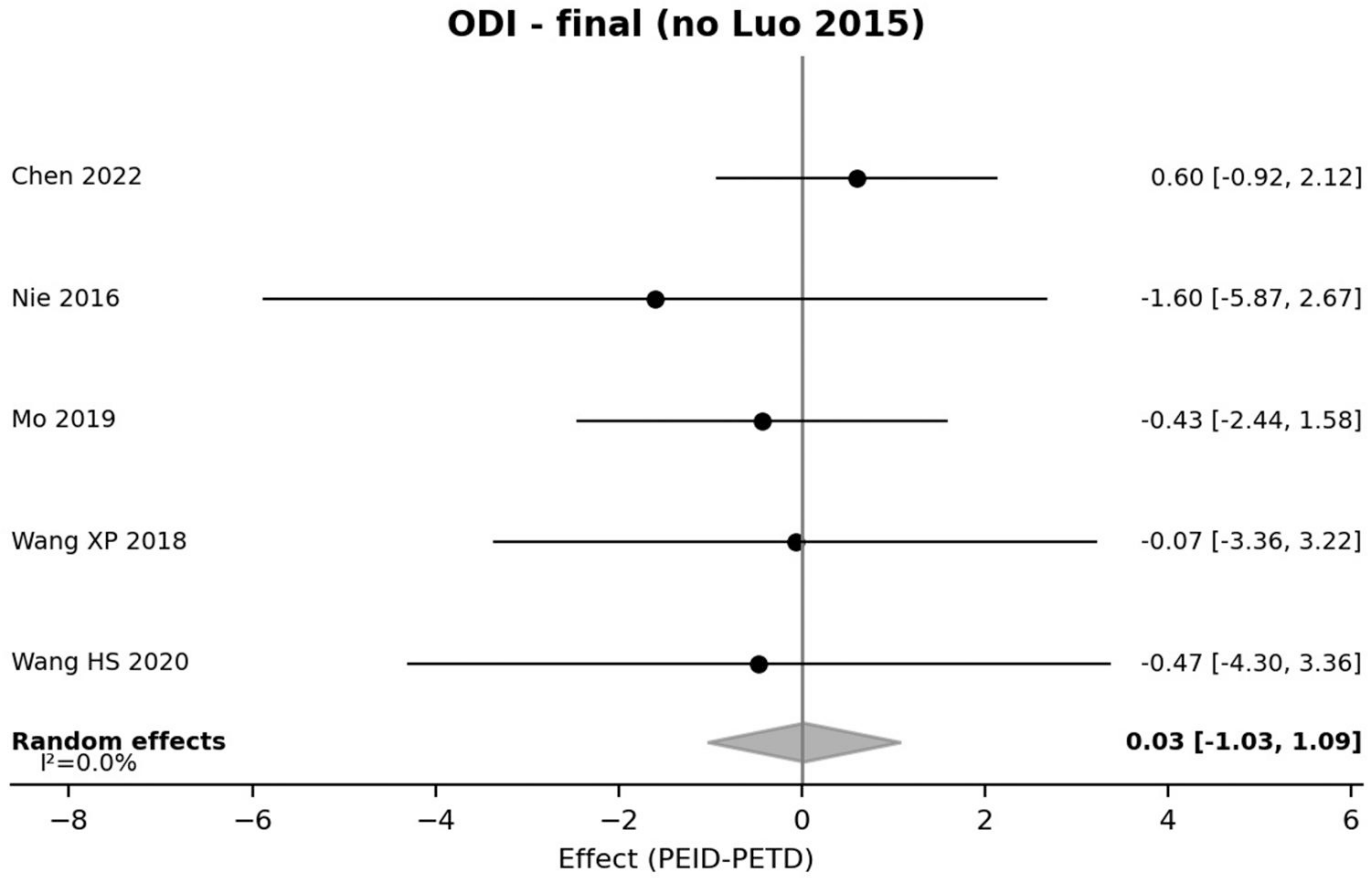
Forest plot comparing ODI scores at final follow-up between PEID and PETD.

For ODI, no statistically significant difference was observed at 1 month (MD = −9.89, 95% CI −29.62–9.85; I2 = 99.2%), 3 months (MD = −1.83, 95% CI −4.11–0.45; I2 = 98.3%; Figure 11), 6 months (MD = −4.43, 95% CI −10.90–2.04; I2 = 99.5%), or final follow-up (MD = 0.03, 95% CI −1.03–1.09; I2 = 0%; Figure 12). Sensitivity analysis excluding Shangguan 2022 for ODI at 3 months did not materially alter the interpretation (MD = −2.40, 95% CI −7.56–2.76; I2 = 97.9%).

### 3.5 Global clinical success and safety outcomes

**Figure 13:**
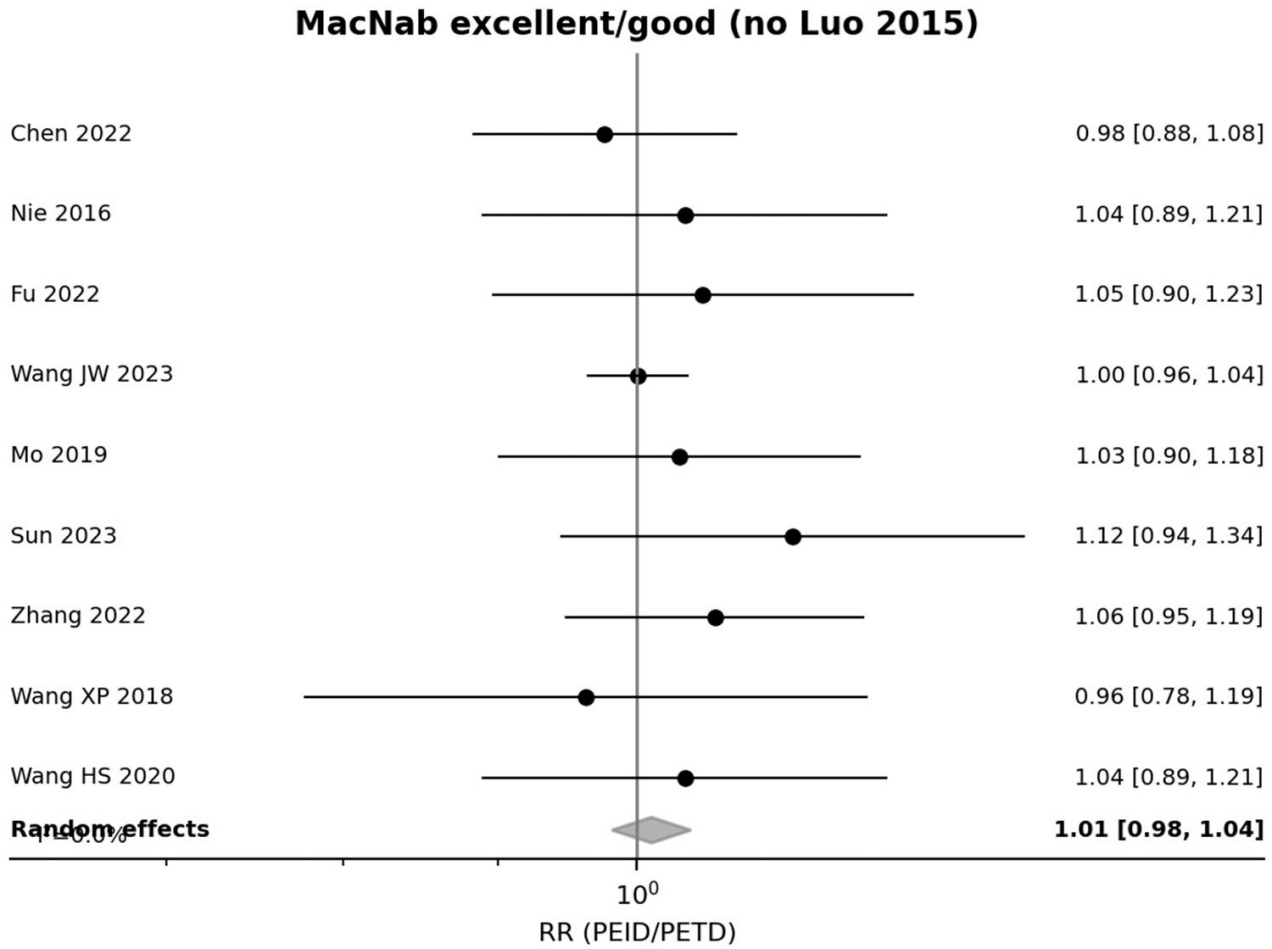
Forest plot comparing modified MacNab excellent/good rate between PEID and PETD using risk ratios.

Nine studies reported extractable modified MacNab excellent/good outcomes. The pooled result showed no statistically significant difference between PEID and PETD (RR = 1.01, 95% CI 0.98–1.04; I2 = 0%; Figure 13). Exploratory with-Luo sensitivity analysis yielded a nearly identical interpretation. Studies that did not report extractable MacNab categories or excellent/good event counts were excluded from this outcome-specific analysis.

**Figure 14:**
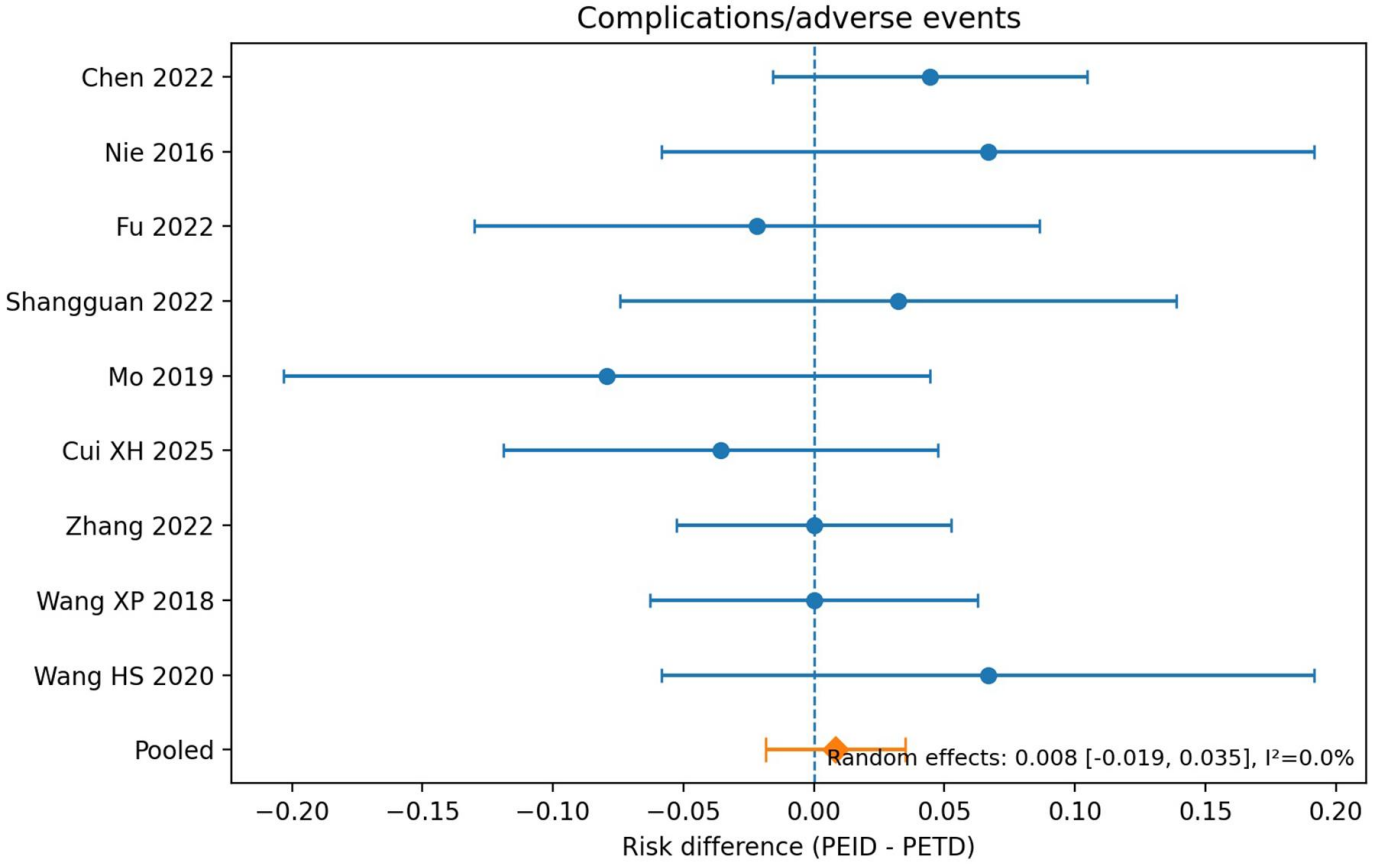
Forest plot comparing complications/adverse events between PEID and PETD using risk differences.

Nine studies reported extractable overall complications or adverse events. The pooled risk difference showed no statistically significant difference between the two approaches (RD = 0.008, 95% CI −0.019–0.035; I2 = 0%; Figure 14). Reported events included dysesthesia, neuropathic pain, dural tear, infection, residual nucleus pulposus, nerve root injury, recurrence, and reoperation when available. Because complication reporting was inconsistent and event numbers were small, safety findings should be interpreted cautiously. Structured safety outcomes are summarized in Supplementary Table S6.

### 3.6 Certainty of evidence

The certainty of evidence was downgraded for several outcomes because of risk of bias, inconsistency, imprecision, and limitations in outcome reporting. Operative time and fluoroscopy frequency were downgraded because of very high heterogeneity. MacNab outcomes were downgraded because of subjective outcome assessment and incomplete blinding. Safety outcomes were downgraded because of small event numbers and inconsistent reporting. A summary of findings is shown in Table 1, and the full GRADE evidence profile is provided in Supplementary Table S5.

**Table 1:** Summary of findings and GRADE assessment for the main outcomes.

Outcome	Participants (studies)	Pooled effect	Certainty	Main reasons for downgrading
Operative time	957 (12 studies)	MD = −17.07 min (95% CI−26.05 to −8.09)	Low	Downgraded for risk-of-bias concerns and very serious inconsistency (I2 = 98.7%).
Fluoroscopy time (seconds)	151 (2 studies)	MD = −3.70 s (95% CI−8.01 to 0.61)	Very low	Downgraded for inconsistency, imprecision, and limited number of studies.
Fluoroscopy frequency (counts)	806 (10 studies)	MD = −8.94 counts (95% CI −11.40 to −6.48)	Low	Downgraded for risk-of-bias concerns and very serious inconsistency (I2 = 99.6%).
Hospital stay	725 (9 studies)	MD = 0.38 days (95% CI−0.36 to 1.12)	Very low	Downgraded for inconsistency and imprecision because the confidence interval crossed the line of no effect.
Bed rest time	485 (7 studies)	MD = 3.63 h (95% CI 1.45 to 5.81)	Low	Downgraded for inconsistency and indirectness due to varied mobilization protocols.
VAS leg pain	Time-point-specific analyses	No consistent significant difference across follow-up time points	Low	Downgraded for inconsistency and imprecision; follow-up schedules varied across studies.
ODI	Time-point-specific analyses	No consistent significant difference across follow-up time points	Low	Downgraded for inconsistency and imprecision; follow-up schedules varied across studies.
MacNab excellent/good rate	719 (9 studies)	RR = 1.01 (95% CI 0.98 to 1.04)	Low	Downgraded for risk-of-bias concerns and subjectivity of the outcome assessment.
Complications/adverse events	689 (9 studies)	RD = 0.008 (95% CI−0.019 to 0.035)	Low	Downgraded for imprecision and inconsistent adverse-event reporting.

## 4 Discussion

This updated systematic review and meta-analysis provides a focused synthesis of randomized evidence comparing PEID and PETD for single-level L5/S1 LDH (12–24). After reconstruction of the search strategy, full-text verification, and data extraction, the revised analysis suggests that PEID may be associated with shorter operative time and reduced fluoroscopy frequency, although the magnitude of these perioperative effects remains uncertain because of very high between-study heterogeneity. However, postoperative pain relief, functional recovery, global clinical success according to the modified MacNab criteria, and safety outcomes were generally comparable between approaches.

The perioperative pattern is clinically plausible. At the L5/S1 level, PETD may be technically affected by a high iliac crest, hypertrophic transverse process, foraminal stenosis, facet obstruction, and a steep puncture trajectory, all of which can increase puncture difficulty, operative time, and fluoroscopy use (4, 5). By contrast, PEID uses the relatively wider L5/S1 interlaminar window and may allow more direct access to intracanalicular fragments in selected patients (2, 4).

Substantial heterogeneity was observed for several perioperative outcomes, particularly operative time, fluoroscopy frequency, hospital stay, bed rest time, and ODI at some follow-up points. This heterogeneity is likely multifactorial. First, surgeon experience and learning curves may strongly influence operative time and fluoroscopy use. Second, anatomical characteristics such as high iliac crest, L5 transverse process hypertrophy, foraminal stenosis, and disc migration may affect PETD and PEID differently. Third, the included studies differed in anesthesia protocols, perioperative mobilization strategies, and reporting units. Fourth, some studies focused on specific subtypes, such as axillary herniation or high-iliac-crest L5/S1 disc herniation, which may not be fully comparable with unselected L5/S1 LDH populations.

To reduce avoidable methodological heterogeneity, we separated fluoroscopy exposure into exposure duration in seconds and frequency counts, standardized bed rest time to hours, and analyzed VAS and ODI according to specific follow-up time points. The exclusion of Luo 2015 from the main RCT-only synthesis and the exploratory with-Luo sensitivity analyses did not materially change the main interpretation. Analysis excluding Shangguan 2022 for segment-specific uncertainty also did not change the interpretation. Nevertheless, because heterogeneity remained high for several outcomes, the magnitude of pooled perioperative effects should be interpreted cautiously.

The interpretation of MacNab outcomes also changed after re-extraction. The modified MacNab criteria represent a global categorical assessment that incorporates symptom relief, functional recovery, residual symptoms, and patient satisfaction. In surgical trials, MacNab assessment may be influenced by lack of blinding, perioperative experience, early recovery, and patient expectations. Therefore, MacNab outcomes should be interpreted alongside continuous patient-reported outcomes such as VAS and ODI. In the revised analysis, MacNab excellent/good rates were comparable between approaches, consistent with the generally similar VAS and ODI findings.

Most included trials were conducted in China, which should be considered when interpreting generalizability. This distribution may reflect the high clinical adoption, large case volume, and active academic output of endoscopic spine surgery in China. However, anatomical demographics, surgical training paradigms, institutional volume, surgeon learning curves, perioperative protocols, and healthcare systems may differ across regions. Therefore, these findings may be most directly applicable to high-volume endoscopic spine settings, and future multicenter randomized trials from diverse healthcare environments are needed.

From a practical surgical perspective, PEID may be advantageous when the L5/S1 transforaminal corridor is restricted by high iliac crest, narrow foramen, facet obstruction, or a difficult puncture trajectory, particularly for central or paracentral intracanalicular fragments accessible through the interlaminar window. PETD may remain appropriate for foraminal or extraforaminal components, selected migrated fragments, or settings in which the surgeon has greater familiarity with the transforaminal approach. Surgical approach selection should therefore be individualized according to anatomy, herniation morphology, and surgeon expertise rather than assuming uniform superiority of one technique.

Several limitations should be acknowledged. First, several included trials did not provide detailed sequence generation or allocation concealment methods, and these studies were judged conservatively in RoB 2 and GRADE. Second, Luo 2015 was excluded from the final RCT-only synthesis because true randomized allocation could not be verified from the full-text methods; exploratory with-Luo analyses were reported only to demonstrate robustness and were not used to support the main conclusions. Third, one included data source was a publicly available master’s thesis report, which was retained because it provided verifiable full-text numerical data for an eligible randomized comparison, not because any unpublished raw data were used (19). For Mo 2019, outcome data were extracted using as-treated sample sizes because one patient crossed over intraoperatively and the report did not provide outcome data according to the original randomized allocation. Use of as-treated rather than intention-to-treat data may introduce bias. An intention-to-treat analysis or allocation-based sensitivity analysis was not possible based on the available published data. Fourth, blinding of surgeons and patients was generally infeasible, and outcome assessor blinding was rarely reported. Fifth, substantial heterogeneity remained for several perioperative outcomes despite unit standardization and time-point-specific analyses. Sixth, most included studies were conducted in China, which may limit generalizability to other healthcare systems and surgical training environments.

## 5 Conclusions

For single-level L5/S1 lumbar disc herniation, both PEID and PETD appear to be effective treatment options. The updated evidence suggests that PEID may be associated with shorter operative time and reduced fluoroscopy frequency compared with PETD, but the magnitude of these perioperative effects remains uncertain because of very high between-study heterogeneity. However, postoperative pain relief, functional recovery, modified MacNab excellent/good outcomes, and safety profiles appear broadly comparable between approaches. Given the methodological limitations, geographic concentration of studies, and substantial heterogeneity in several outcomes, these findings should be interpreted cautiously, and procedure selection should remain individualized according to anatomy, access corridor, herniation morphology, and surgeon experience.

## Data Availability

The original contributions presented in the study are included in the article/Supplementary Material, further inquiries can be directed to the corresponding author.

## Supplementary material

Supplementary Table S1 Complete database-specific search strategies.

Supplementary Table S2 Full-text assessment and exclusion reasons.

Supplementary Table S3 Characteristics of included studies.

Supplementary Table S4 RoB 2 domain-level judgments and justifications.

Supplementary Table S5 GRADE evidence profile.

Supplementary Table S6 Structured safety outcomes.

Supplementary Table S7 Sensitivity analyses and prediction intervals.
