# Supplementary figures and images for: Percutaneous endoscopic interlaminar discectomy vs. percutaneous endoscopic transforaminal discectomy for L5/S1 lumbar disc herniation: a systematic review and meta-analysis of randomized controlled trials
Source: Front Surg. 2026 Jul 14;13:1853050. doi: 10.3389/fsurg.2026.1853050 (PMC13405533; doi:10.3389/fsurg.2026.1853050)

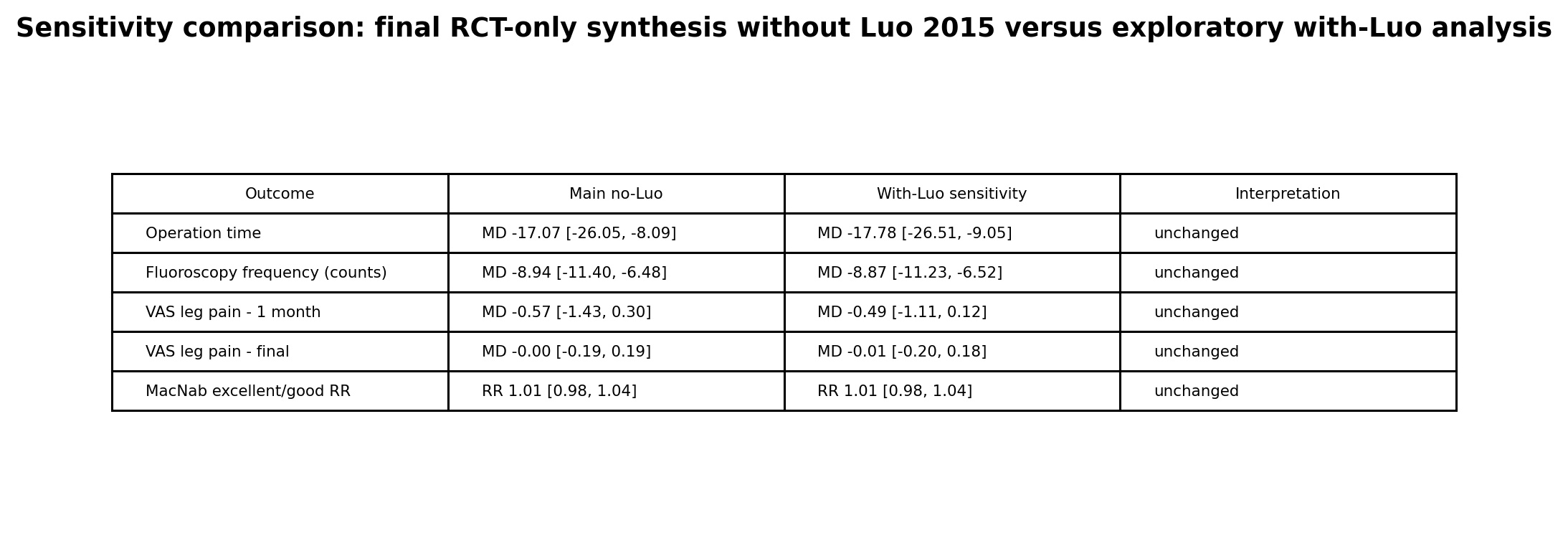

Supplement: Supplementary file 9 [file Image1.jpeg]

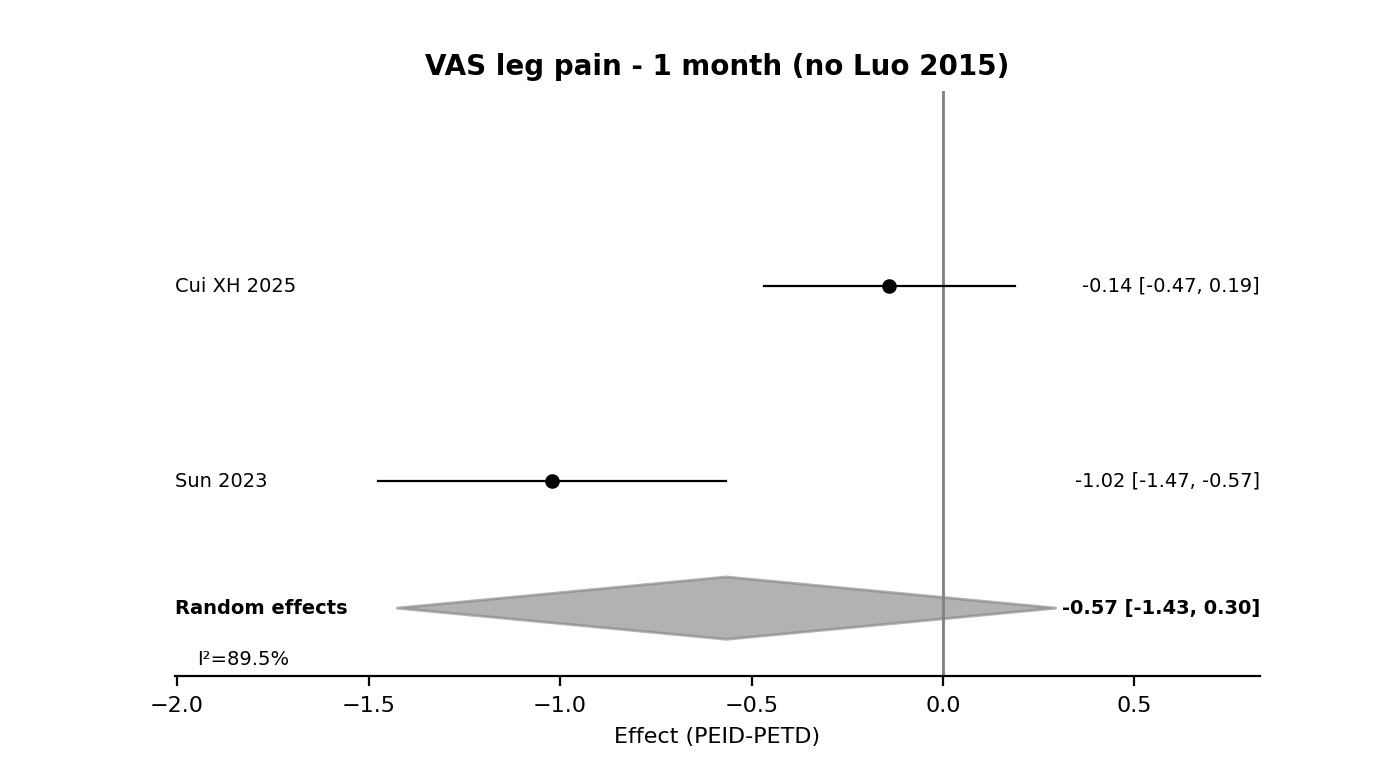

Supplement: Supplementary file 10 [file Image2.jpeg]

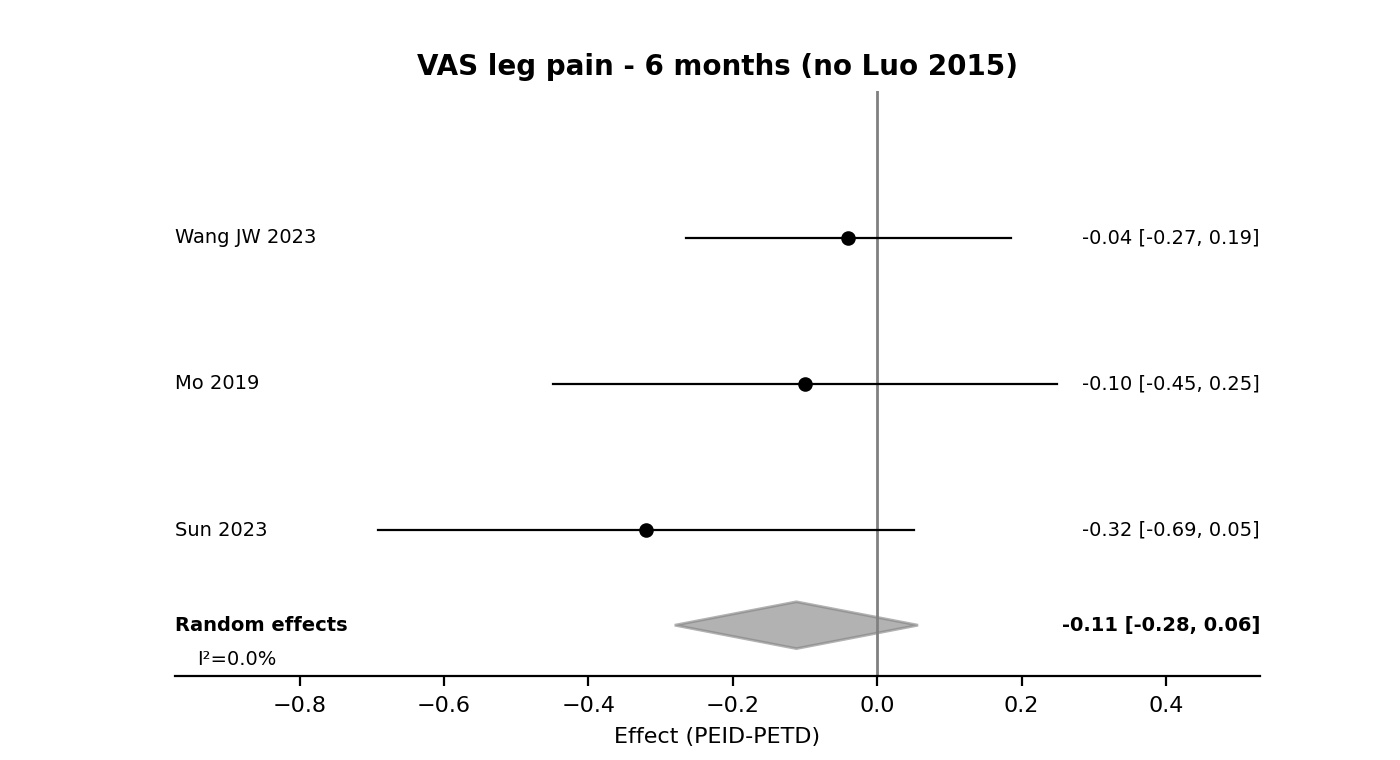

Supplement: Supplementary file 11 [file Image3.jpeg]

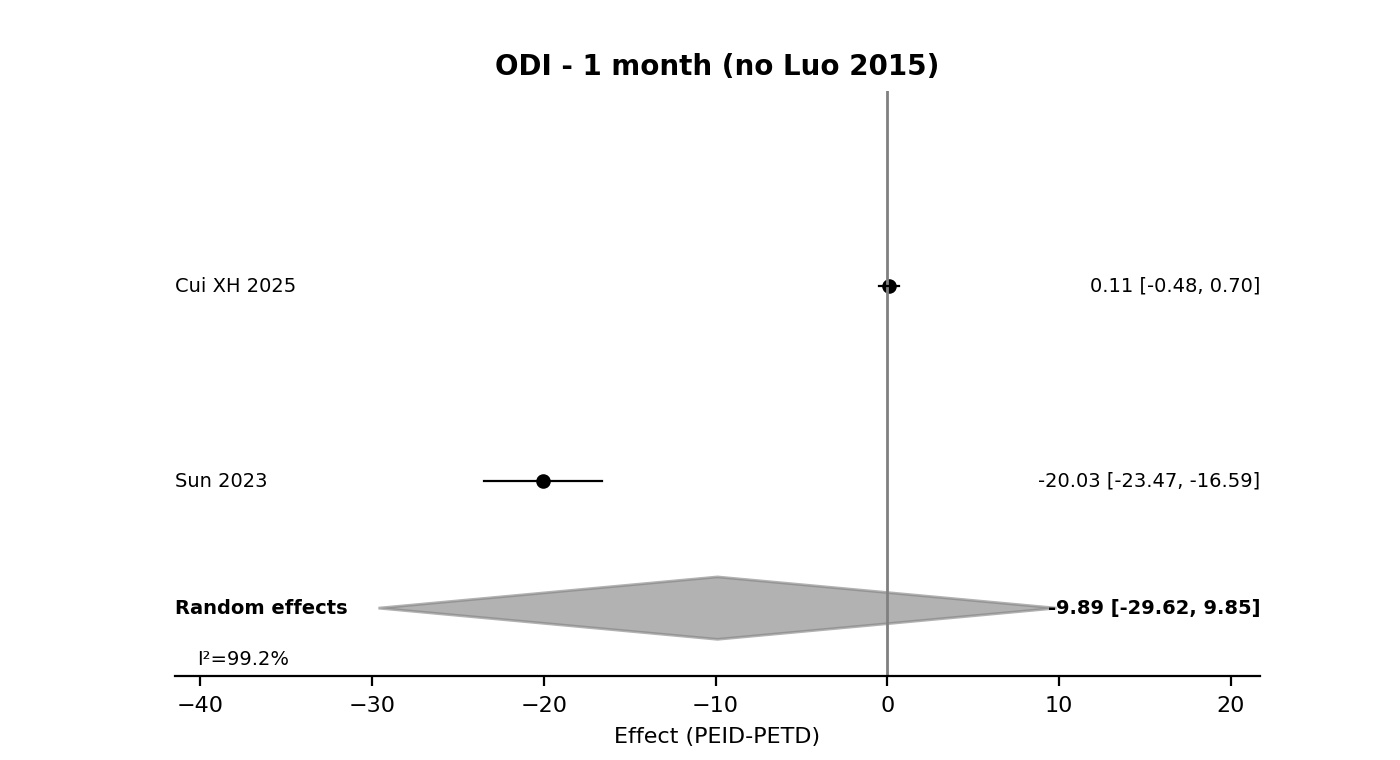

Supplement: Supplementary file 12 [file Image4.jpeg]

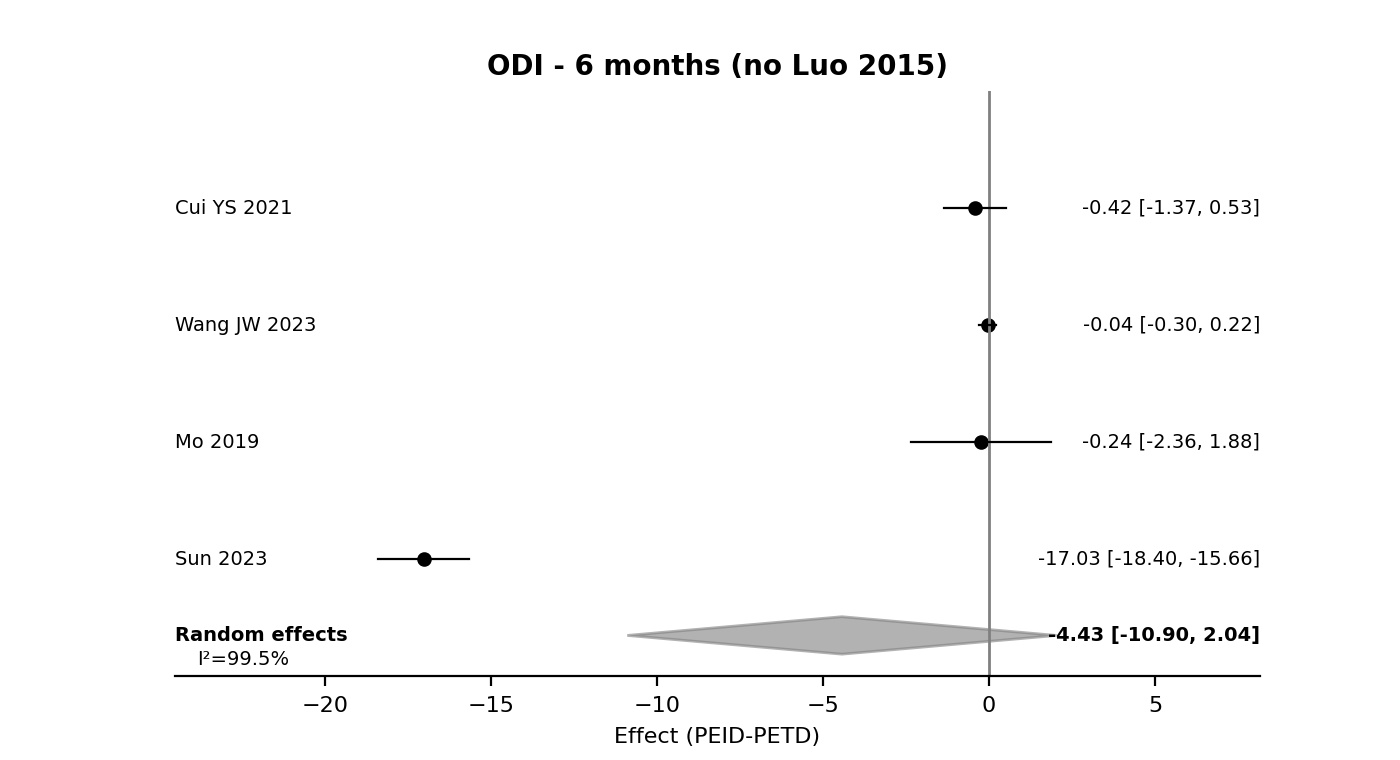

Supplement: Supplementary file 13 [file Image5.jpeg]
